# Revolutionizing healthcare information systems with blockchain

**DOI:** 10.3389/fdgth.2023.1329196

**Published:** 2024-01-11

**Authors:** Dongfang Wu, Yichen Wang

**Affiliations:** Global Health Research Center, Duke Kunshan University, Kunshan, China

**Keywords:** healthcare information system (HIS), blockchain, data integrity, traceability, revolution

## Introduction

The healthcare industry has long grappled with the challenges of ensuring the integrity, security, and traceability of patient data. Traditional healthcare information systems often face issues like data falsification, single points of failure, and a lack of data traceability. In recent years, blockchain technology has emerged as a promising solution to address these problems. This article explores the potential of using blockchain technology to construct a robust and reliable healthcare information system.

## Blockchain technology in healthcare

Blockchain technology, which underlies cryptocurrencies like Bitcoin, offers three key characteristics that can revolutionize healthcare information systems:

1. Consensus Mechanism: Blockchain utilizes consensus mechanisms such as Proof of Work or Proof of Stake to incentivize network participants (nodes) to contribute valid data. This ensures the integrity of the data and addresses the challenge of potential data falsification (1).
2. Distributed Ledger: The distributed nature of blockchain eliminates the possibility of a single point of failure. Multiple nodes simultaneously perform identical tasks, enhancing the system's reliability (2).
3. Chain Structure: The chain structure of blockchain greatly enhances the traceability of healthcare information, from its generation to workflow (3).

## Practical applications and advantages

Several blockchain-based systems have already been developed for healthcare information management:

1. MedChain System: MedChain introduces a novel incentive mechanism focused on data validation, which ensures consistency and sustainability, reducing the risk of data falsification (4).
2. LifeCODE.ai: This system employs a distributed ledger to prevent single points of failure and ensures the reliable and steady recording of data, even on mobile devices (5).
3. Polygon: As an Ethereum sidechain system, Polygon boasts a strong chain structure and provides robust traceability capabilities in data management (6).

Numerous researchers have demonstrated the advantages of using blockchain in healthcare information systems. Experimental approaches have shown the feasibility and efficacy of this technology in enhancing the storage and management of healthcare data (7).

## Challenges to overcome

While blockchain technology offers substantial benefits, there are challenges that must be addressed when adopting it in healthcare information systems:

1. Transaction Speed: Blockchains, such as Bitcoin, often have unsatisfying transactions per second (TPS) and may experience congestion during the transmission of healthcare information, requiring higher throughput (8). In response to this challenge, Layer 2 scaling solutions like Lightning Network for Bitcoin or Sidechains for Ethereum may hold promise as resolution (9, 10).
2. Transaction Fees: Every transaction of healthcare information on a blockchain network requires a fee, making it essential to reduce these costs to ensure the economic viability of the system (11). Some blockchains have proposed solutions to reduce transaction fees, exemplified by Ethereum's EIP-1559 pricing scheme and Arbitrum's Optimistic Rollups mechanism (12, 13).
3. Off-Chain Data Compatibility: The immaturity of off-chain aggregation mechanisms presents difficulties in synchronizing off-chain healthcare information with the blockchain, hindering data consistency (14). In this regard, Chainlink offers an Oracle solution to efficiently transmit off-chain data onto the blockchain, which enables the synchronization and consistency of on-chain and off-chain healthcare information (15).

## The way forward: Blockchain 3.0 and Internet Computer Protocol

To overcome these challenges and construct a more advanced healthcare information system on the blockchain, it is crucial to explore the potential of Blockchain 3.0 technology, such as Internet Computer Protocol. Blockchain 3.0 aims to enhance scalability, reduce transaction costs, and improve data compatibility, making it a promising candidate for the future of healthcare information systems.

## Conclusion

Blockchain technology presents a compelling solution to the long-standing challenges of healthcare information management. While there are obstacles to overcome, the benefits of enhanced data integrity, security, and traceability make the adoption of blockchain in healthcare a worthwhile endeavor. With the ongoing development of Blockchain 3.0 technologies like Internet Computer Protocol, the future of healthcare information systems holds great promise.
